# Direct Synthesis of Novel and Reactive Sulfide-modified Nano Iron through Nanoparticle Seeding for Improved Cadmium-Contaminated Water Treatment

**DOI:** 10.1038/srep24358

**Published:** 2016-04-20

**Authors:** Yiming Su, Adeyemi S. Adeleye, Yuxiong Huang, Xuefei Zhou, Arturo A. Keller, Yalei Zhang

**Affiliations:** 1 State Key Laboratory of Pollution Control and Resources Reuse, Tongji University, Shanghai 200092, China; 2 Bren School of Environmental Science & Management, University of California, Santa Barbara, 3420 Bren Hall, CA 93106, USA; 3 University of California Center for Environmental Implications of Nanotechnology, Santa Barbara, California, USA; 4 Key Laboratory of Yangtze Water Environment for Ministry of Education, Tongji University, Shanghai 200092, China

## Abstract

Magnetic sulfide-modified nanoscale zerovalent iron (S-nZVI) is of great technical and scientific interest because of its promising application in groundwater remediation, although its synthesis is still a challenge. We develop a new nanoparticle seeding method to obtain a novel and reactive nanohybrid, which contains an Fe(0) core covered by a highly sulfidized layer under high extent of sulfidation. Syntheses monitoring experiments show that seeding accelerates the reduction rate from Fe^2+^ to Fe^0^ by 19%. X-ray adsorption near edge structure (XANES) spectroscopy and extended X-ray absorption fine structure analyses demonstrate the hexahedral Fe-Fe bond (2.45 and 2.83 Å) formation through breaking down of the 1.99 Å Fe-O bond both in crystalline and amorphous iron oxide. The XANES analysis also shows 24.2% (wt%) of FeS with bond length of 2.4 Å in final nanohybrid. Both X-ray diffraction and Mössbauer analyses further confirm that increased nanoparticle seeding results in formation of more Fe^0^ crystals. Nano-SiO_2_ seeding brings down the size of single Fe^0^ grain from 32.4 nm to 18.7 nm, enhances final Fe^0^ content from 5.9% to 55.6%, and increases magnetization from 4.7 to 65.5 emu/g. The synthesized nanohybrid has high cadmium removal capacity and holds promising prospects for treatment of metal-contaminated water.

Nanoscale zerovalent iron (nZVI) is becoming increasingly popular as a potential new approach for heavy metal pollution treatment^1^,^2^,^3^, and field-based pilot case studies have been reported^4^,^5^. However, there are still two main drawbacks with nZVI technology: one is the limited removal capacity for some particular metal ions, e.g. Cd^2+^ (removal capacity is only 40 mg/g^6^, compared with >1600 mg/g for Pb^2+^)^7^. The other challenge is the poor chemical stability of metal-nZVI mixture as both cations and anions in groundwater can affect the removal performance. According to previous studies, Cl^−^ can significantly inhibit the Cd^2+^ immobilization by nZVI;^8^ NO_3_^−^ will result in the remobilization of Pb^2+^;^9^ HCO_3_^−^ and Ca^2+^ greatly decreases the chemical stability of Uranium-nZVI mixture^10^. Even for Fe_3_O_4_ or multiwalled carbon nanotube modified nZVI, SO_4_^2−^, HCO_3_^−^ and NO_3_^−^ also lead to a decrease in Cr(VI) removal efficiency^11^,^12^. Improving the metal removal capacity and enhancing the chemical stability of metal-nZVI mixture are of great importance for the practical use of nZVI in industrial wastewater treatment and groundwater remediation.

Recent advance in this area involves incorporation of sulfur into nZVI (sulfidation), to make composites such as Fe^0^/FeS and sulfide-modified nanoscale zerovalent iron (S-nZVI), for improving metal removal capacity and enhancing the chemical stability of metal-(S-nZVI) mixtures^13^,^14^,^15^,^16^. According to our previous study^13^, although high sulfidation is preferred, a high dosage of sodium dithionite in the reductant (to form S-nZVI) suppresses the formation of magnetic Fe^0^/FeS nanohybrids. These Fe^0^-based nanohybrids endow the nanomaterials with high adsorption capacity^7^, strong reducing capacity^14^,^17^ and the capability of carrying out Fenton-like reactions^18^. Hence, more fundamental research is necessary for synthesizing of Fe^0^-based nanohybrids, further exploring the metal removal performance of S-nZVI, and for strengthening its practical application.

To obtain nanoparticles, nucleation and crystal growth are crucial steps. Nucleation is a process of nuclei formation, providing templates for subsequent crystal growth^19^. It can be classified into homogeneous and heterogeneous, with the latter one occurring more easily due to lowered surface free energy resulting from the stable presence of active centers^20^,^21^. Among many heterogeneous nucleation methods, seeded nucleation is typical^21^,^22^, and is found to be necessary for reduction processes of some complexes^23^,^24^. To the best of our knowledge, no effort has been made to systematically investigate the formation of Fe^0^/FeS nanohybrid through homogeneous or heterogeneous nucleation.

Furthermore, the structural evolution of S-nZVI during its synthesis is interesting, and is important for its performance. Su *et al*.^13^ reported that due to the addition of dithionite, the core-shell structure of pristine nZVI evolved into a sphere covered by a flake-like structure, the typical structure of transition metal sulfide^25^. While there are many studies on homogeneous nucleation^26^,^27^, studies on nanoparticles forming a flake-like structure through heterogeneous nucleation are limited. Although impurities, seeds as an example, are able to facilitate nucleation^28^, they are also able to retard the entrance of crystalline ions from solution onto certain sites of the evolving crystal, which may result in a change of the growth pattern and final morphology of the synthesized nanomaterials^29^. Here, we focused on the effect of nanoparticle seed types and concentrations on the heterogeneous nucleation process and structural evolution of S-nZVI particles during sulfidation, which are of great importance, but have not been studied before.

In this study, our main objectives include developing a new one-pot method to synthesize a unique nanoiron-hybrid material, determining the mechanisms of synthesis that results in this new material. Seeding was done using nano-SiO_2_, nano-TiO_2_ and nano-Al_2_O_3_. Transmission electron microscopy (TEM) with an energy-dispersive X-ray spectroscopy (EDS) probe was employed to monitor the structure evolution during particle synthesis. X-ray diffraction (XRD) was used to determine the size of crystals and the composition of final particles. Vibrating sample magnetometer (VSM) analyses were used to study the magnetic characterization of the different final particles. Mössbauer and X-ray adsorption near edge structure (XANES) measurements were carried out to investigate the compositions in different samples. We also evaluated the removal capacity of S-nZVI seeded with nano-SiO_2_, denominated FeSSi, for different metal ions in a simulated groundwater and wastewater.

## Results and Discussion

### Nucleation process

**Figure 1:**
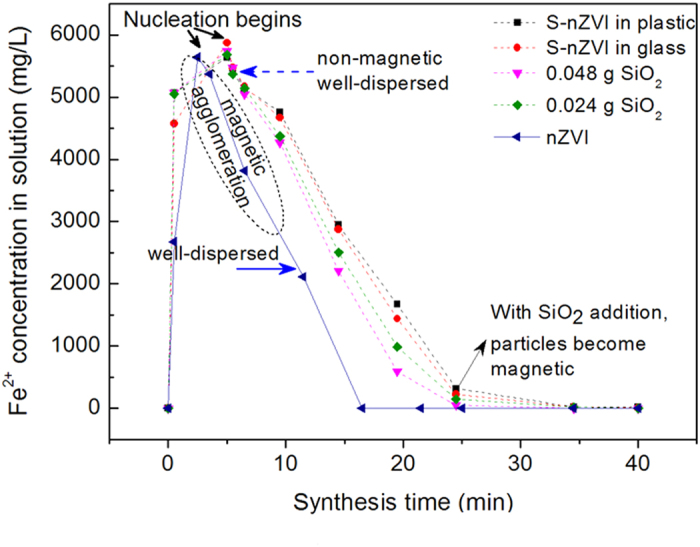
Fe^2+^ trend in FeCl_3_•6H_2_O solution during titration (all the collected samples were passed through a 0.22 um filter.

As can be seen in Fig. 1, the synthesis process involves the reduction of ferric ions to ferrous ions first (equation 1), and then nucleation (equation 2)^13^. The formation of pristine nZVI follows the zero-order reaction model^30^, which is quite interesting. According to the Finke-Watzky 2-step theory, a slow, continuous nucleation and fast autocatalytic growth should be observed, resulting in a sigma plot^22^. However, in the case of nZVI synthesis system without a stabilizer, nZVI agglomerate severely, which means autocatalytic growth is much faster than nucleation. In other words, the reaction rate of eq.1 determines the rate of nanoparticle formation. However, formation of S-nZVI is not well-described by a zero-order reaction model. We did not observe any significant influence of reactor material on nucleation. However, nano-SiO_2_ seeding accelerated iron reduction by about 19%, calculated by comparing the rate between S-nZVI (in glass) and FeSSi (with 0.048 g nano-SiO_2_) systems. Similar trend of Fe^2+^ concentration was also observed in the synthesis system with nano TiO_2_ or nano Al_2_O_3_ addition (Fig. S1).


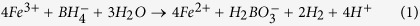



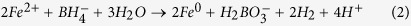


It is noteworthy that the black particles formed in the nZVI system were inherently magnetic, and thus rapidly agglomerated. This indicates the formation of Fe^0^. However, in the S-nZVI system (without nanoparticle seeding), the initial particles were non-magnetic and dispersed well. A small fraction of the particles exhibited weak magnetism at the very end of the synthesis, indicating the formation of a small amount of Fe^0^. Interestingly, when nano-SiO_2_ was seeded into the reductant, although the initial FeSSi particles were non-magnetic, they became strongly magnetic at the later phase of the synthesis.

Agglomeration of nZVI at the beginning is due to the strong magnetic force and the absence of a stabilizer^31^, which can be classified into two categories: electrostatic (charge or inorganic) stabilization and steric (organic) stabilization^32^. With the continuous addition of reductants, the system’s pH becomes alkaline, and the negative surface charge of nZVI imparts electrostatic stabilization^33^. This may be the reason why nZVI particles were well-dispersed at later phase of synthesis.

However, the nucleation process of S-nZVI and FeSSi was different from nZVI. The initial non-magnetic well-dispersed particles formed were probably iron salt clusters. The reduced ferrous ions formed green rust which co-existed with ferric ions during the initial phase of nucleation. Then with continuous addition of reductant, some ferrous and ferric ions were reduced to Fe^0^. Generally, this kind of wet-chemical method uses surfactants or organic coatings to prevent agglomeration^34^,^35^, however, some studies showed that Cl^−^ also can work as stabilizer, providing electrostatic repulsion^36^,^37^. Due to the abundance of Cl^−^ in the present system, the nanoparticles formed were well-dispersed.

### Structure evolution during syntheses

**Figure 2:**
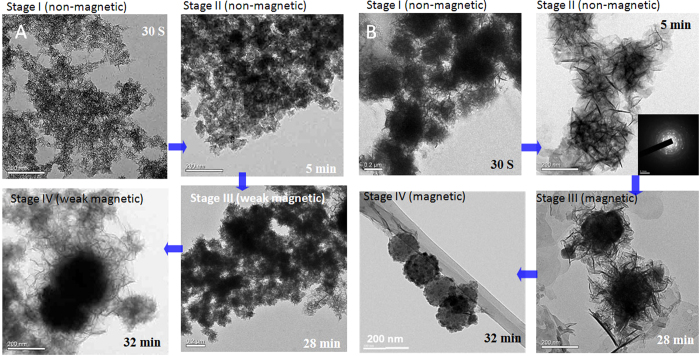
Structure evolution of particles in S-nZVI synthesis (**A**) FeSSi synthesis (**B**) (with seeding of 0.048 g nano-SiO_2_). Time value means the time after nucleation. The scale bar in images represents 200 nm except the one at 32 min in (**B**) (it represents 20 nm).

TEM was employed to study the morphological change of the materials during the synthesis process (Fig. 2). For S-nZVI, the initial particles formed in solution were non-magnetic, whether or not seeding was done. This implies that these particles were not Fe^0^. In the second stage, there is an obvious difference between the systems with nanoparticle seeding and those without it; particles in systems without nanoparticle seeding were amorphous (Fig. 2A) while in the systems with seeding (Fig. 2B) they were heteromorphic (as suggested by the electron diffraction pattern). In the systems with nanoparticle seeding, flake-like structures were observed, suggesting that seeding can lower the surface free energy and facilitate the formation of flake-like structures rather than more compact spherical structures of pristine nZVI. Additionally, particles were non-magnetic in both systems during this stage, and the flake-like structure could be due to the presence of Cl^−^, which may function as a shape controller when incorporated into the iron cluster^38^.

In the third stage, particles in systems with nanoparticle seeding became magnetic; we only observed very weak magnetism in the systems without seeding. Some of the flake-like structures observed in Stage II in the seeded systems were replaced by black spherical nanoparticles, likely Fe^0^ particles, which were responsible for the change in magnetization. However, in systems without seeding, the structures did not change significantly. In the last stage, several particles surrounded by flake-like structure in the seeded systems evolved into particles with a compact core-shell structure (Fig. 2B, Stage IV). Notably, these clear compact structures were only a part of the final materials as some flake-like structures persisted, similar to Stage III in Fig. 2B (also see Fig. S2). EDS analysis indicated the particles had higher content of iron than their flake-like predecessors, and sulfur was present in both systems (Fig. S3)

### Different reduction pathway during synthesis

**Figure 3:**
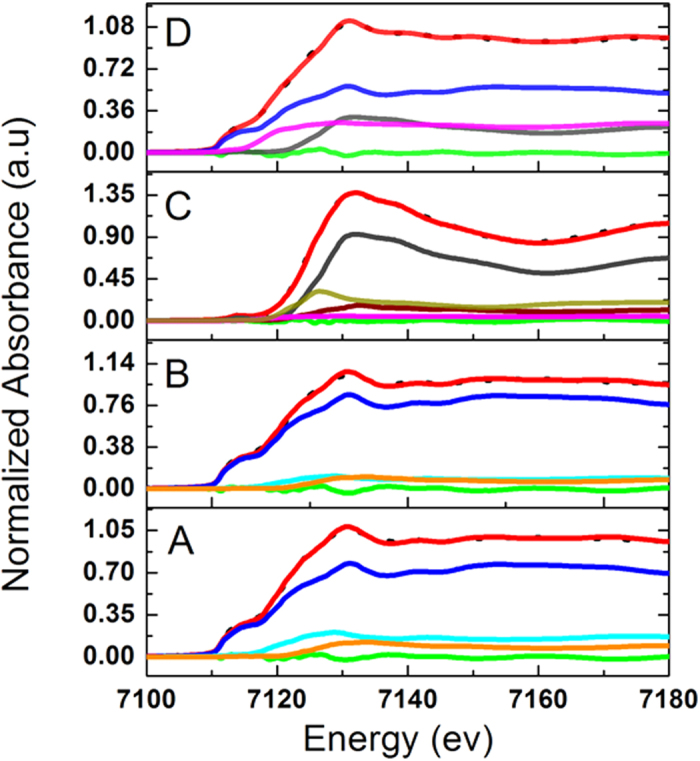
Fe K-edge XANES liner combination fit for nanoparticles collected during synthesis: middle stage (**A**) and final stage (**B**) during nZVI synthesis process; middle stage (**C**) and final stage (**D**) during FeSSi synthesis process (- -, data; 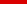, fit; 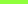, residual; 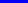, Fe(0); 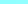, FeO; 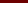, gamma Fe_2_O_3_; 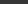, Fe_3_(PO_4_)_2_; 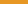, Hematite; 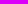, FeS; 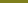, FeSO_4_;). (Fe_3_(PO_4_)_2_ was used to represent the disordered Fe-O bond).

To further investigate the reduction pathway of FeSSi, XANES was employed to analyze the composition of nanomaterials collected at different time intervals from nZVI (Fig. 3a,b) and FeSSi (Fig. 3c,d) synthesis systems. A seen from Fig. 3a,b, collected at middle and final synthesis stages, respectively, the main composition of the particles at both stages is zerovalent iron. This indicates the continuous Fe^0^ nucleation from Fe^2+^ in solution (to form nanoparticles). However, in FeSSi synthesis system, instead of Fe^0^, abundance of iron oxide, both crystalline and amorphous, was initially observed. Then, iron oxide was then reduced to Fe^0^. Meanwhile, FeS was also formed. Liner combination fitting result shows 55.6% and 24.2% of Fe^0^ and FeS, respectively, in final nanohybrid (Table S1), both of which are important for pollutants removal.

Furthermore, Fourier transform magnitude of *K*^3^ weighted Fe *K*-edge extended X-ray absorption fine structure (EXAFS) spectra for FeSSi collected at middle and the end stage of synthesis (Fig. S5) give out more detailed information on structural evolution. The structural parameters of both samples gained by EXAFS analysis are given in Table 1. In accordance with the EXAFS data, it can be observed that the primarily 1.99 Å Fe-O bond^39^,^40^ at middle stage transforms to hexahedral Fe-Fe bond (2.45 and 2.83 Å) and 2.4 Å Fe-S bond with the increasing addition of reductant. Additionally, the Fe-O bond distance decreases to 1.89 Å at the same time. This result directly corroborates our hypothesis that nanoparticulate seeding can facilitate Fe^0^ nucleation and growth under high extent of sulfidizing conditions.

### Crystal structure of nanomaterials through XRD analysis

XRD was employed to characterize the cystal structure of final nanomaterials from different synthesizing systems. X-ray diffractograms reveal that Fe^0^ exists in all the synthesized particles but the crystallinity (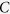) of Fe^0^, which is defined in Eq. 3 41, varies widely:


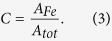


where 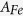 is the peak area of Fe^0^, and 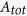 is the total peak area of the diffractogram, including crystalline and non-crystalline peaks. nZVI has the highest C (73.7%), and S-nZVI without nanoparticle seeding has the lowest C (11.2% and 9.5% for glass and plastic reactors, respectively). Seeding with nano-SiO_2_ increased Fe^0^ crystallinity in FeSSi, by up to a factor of 5 compared to S-nZVI (Table 2). Furthermore, nano-TiO_2_ and Al_2_O_3_ can facilitate Fe^0^ crystallization, although not as much as nano-SiO_2_.

**Figure 4:**
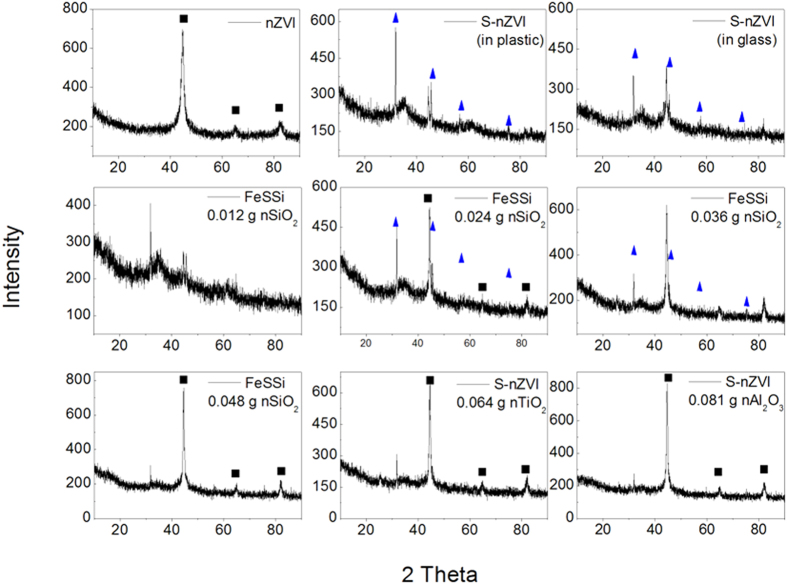
XRD patterns of nZVI, S-nZVI synthesized in plastic and glass beaker, and S-nZVI synthesized with different dosage of seeding nanoparticles. (◼, peaks for Fe^0^; 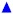, peaks for NaCl).

Some NaCl was observed in all S-nZVI and FeSSi particles (Fig. 4). Na^+^ was contributed by sodium dithionite and sodium borohydride, while the Cl^−^ ions came from the ferric chloride. Sodium and chloride were adsorbed onto the iron cluster, providing the electrostatic repulsion. This explains the well-dispersion of S-nZVI and FeSSi observed during the syntheses.

Further analysis of the XRD diffractogram through Scherer’s formula (equation 4)^42^ can be used to estimate the size of single Fe^0^ crystals in the various synthesized particles:


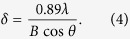


In Eq. 4, 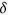 is the size of single crystal size, 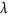 is the wavelength of the Cu*K*_α_ radiation employed (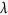 = 0.154 nm), B is the experimentally observed diffraction broadening (in radians), and θ is the Bragg angle. nZVI has the smallest Fe^0^ crystals while S-nZVI (in plastic and glass) has the largest Fe^0^ crystals (Table 2). The size of single Fe^0^ crystal in FeSSi decreases with increasing dosage of nano-SiO_2_. Nano-TiO_2_ and nano-Al_2_O_3_ also facilitated the formation of single Fe^0^ crystal at a size to similar to that of nano-SiO_2_ seeds.

In accordance with classical nucleation theory^20^, the total free energy (Δ*G*) of a spherical particle (radius = r) for homogeneous nucleation is the sum of surface free energy (*γ*) and the bulk free energy (Δ*G*_*v*_):


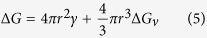


where bulk free energy is defined as:


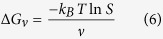


Here 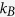 is Boltzmann’s constant, *T* is temperature, *S* is the solution supersaturation and 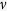 is molar volume.

Given that surface free energy and bulk free energy are positive and negative, respectively, there is a maximum value for the total free energy (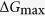), illustrated by Fig. S4. By differentiating 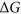 with respect to r and setting it to 0, the critical r value (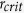), at which 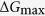 is achieved can be obtained (equation 7)^20^. 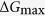
can be subsequently calculated as shown in eq. 8. The critical radius is considered as the minimum size at which a particle can avoid re-dissolution. In other words, below this size, crystal growth is unfavorable.


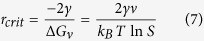



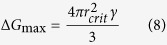


Dithionite may act as an ionic impurity, which can retard the incorporation of Fe^2+^ ions into the crystallization sites of Fe^0^ 43. In addition, dithionite may compete with Fe^2+^ or Fe^3+^ to form precipitates^13^. Both influences contribute to the increase of *γ*, and thus inhibit the nucleation process. However, some nucleation still occurred in the modified particles as indicated by the XRD diffractograms. Considering that the same reductant (NaBH_4_) concentration was used in all syntheses, and that the same initial/final ionic iron concentration was measured, when nucleation was restricted, crystal growth was favorable resulting in a larger crystal size^28^. Hence, the single Fe^0^ crystal size of S-nZVI (synthesized in plastic and glass container) was much larger than for nZVI (see Table 2).

The slight difference in crystal size between S-nZVI prepared in plastic and glass surfaces is likely due to differences in the affinity between nuclei and active centers in the different reactors. Unlike homogeneous nucleation, crystals grown on support surfaces are no longer spherical, but do form in a semi-spherical at a contact angle 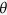 with the support^20^. Differences in affinity between nuclei and active centers cause the surface free energy to decrease to a different extent. As a result, the maximum total free energy (equation 9)^20^ for heterogeneous nucleation decreases correspondingly.


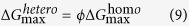


Furthermore, *ϕ* (defined in equation 10), which ranges from 0 to 1, will increase as a function of *θ*.


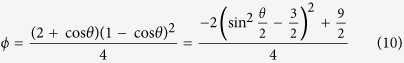


In this study, θ was 35° for glass and 103° for plastic. The glass support system will therefore have a lower 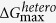. Accordingly, nucleation occurs more easily in a glass support surface than in plastic.

Furthermore, when SiO_2_ is present in the system, the nucleation rate (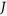) can be accelerated according to eq. 11 44, given the significant decrease in *γ*^21^.


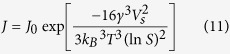


where 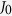 is the nucleation constant and *V*_*s*_ is molecular volume. With all other parameters constant, nucleation rate increases with increased dosage of nano-SiO_2_ (Table 2, and also confirmed by the trend of Fe^2+^ presented in Fig. 1). Since the total amount of iron is constant during synthesis, an accelerated nucleation rate inevitably restricts crystal growth, leading to smaller crystal sizes (Table 2).

However, it is difficult to determine the value for 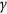, and so far it remains unknown for small nuclei^22^. The factors that influence 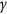 include surface density of surface monomers, ratio of open sites on a surface monomer to that of free monomers, and temperature^45^. Additionally, the energy required to initiate a step on the crystal surface and the free-energy barrier for an adsorbed solute molecule to be incorporated into the kink site of the crystal are crucial for crystal growth^43^. It is very likely that different seeding nanoparticles have different levels of impact on 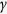 and those two energy-requiring steps, and thus affect the rate of nucleation and crystal grow to different extents.

### Magnetic characteristics of different nanomaterials

For iron, the superparamagnetic region of zero coercivity continues to approximately 10 nm^46^. The peak in coercivity (Hc), which coincides with the development of multiple magnetic domains, was reported to occur around 100 nm^46^. For nZVI it is reasonable to observe a high Hc (604.08 Oe, in Table 3) given that the average size is around 60 nm. However, VSM analyses suggest that S-nZVI is probably ferrimagnetic, which agrees well with the low Fe^0^ crystallinity found via XRD analysis (Table 2). In addition, Hc of FeSSi increases with nano-SiO_2_ seeding. The increase in Hc is probably due to increase in Fe^0^ content (confirmed using Mössbauer analysis) and the number of atoms on surface of the particles^47^ as the size of Fe^0^ crystals decreases (from 28.0 to 18.7 nm) with increasing nano-SiO_2_ seeding (from 0.012 g to 0.048 g), as shown by XRD analysis.

**Figure 5:**
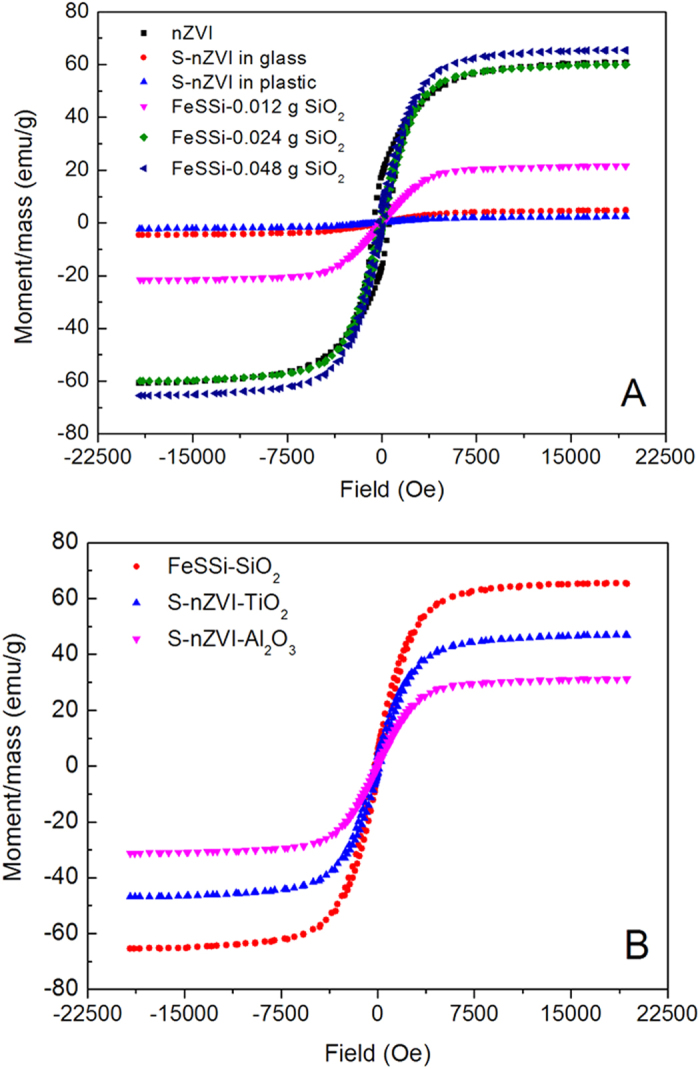
Hysteresis loop of different nanomaterials from systems with (**A**) different concentrations of nano-SiO_2_ and (**B**) different nanoparticle seeding.

In addition, magnetization (M) increases substantially with increase in SiO_2_ dosage (Table 3, Fig. 5A). High M can result in particles that are easy to remove from suspension with a simple magnetic field. According to the literature^46^, M is relatively difficult to change without a change in synthesis procedure. It is, thus, very much likely that the increase in nano-SiO_2_ seeds caused the increase of M in FeSSi. This agrees well with the XRD results that indicate an increase in Fe^0^ crystallinity with increasing nano-SiO_2_ dosage. Increased Fe^0^ in FeSSi might be the primary reason for enhancement of M, compared to S-nZVI.

In accordance with conventional theory^46^,^48^, 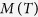
can be calculated using equations from eq. 12, 13, 14, 15:


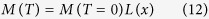



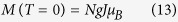



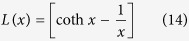



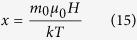


where *N* is the number of magnetic atom per volume, *g* is the Lande sepctroscopic g-factor, *J* is the total angular momentum, 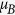
is the Bohr magnetron, 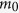
is the classic atomic moment 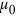
is the magnetic constant, *k* is Boltzmann constant, *H* is the applied magnetic field.

Since eqs 14 and 15 are equal for all the FeSSi particles, 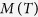
is determined by 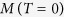 for each particle. The difference in 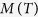 among all these S-nZVI/FeSSi particles depends largely on *N*, which is directly related to the size of a single Fe^0^ crystal—as crystal size decreases *N* increases. This agrees with our previous observation that increased nano-SiO_2_ seeding increases *M*, as the single crystal size of Fe^0^ declines.

The retentivity (Mr) of the final nanomaterials is different as well (Table 3). Although Mr is quite high for nZVI, it decreases to below 1 emu/g for S-nZVI (without nanoparticle seeding) due to the lack of Fe^0^ crystals. However, with increased nanoparticle seeding, the Mr of FeSSi increases as well. FeSSi derived from system with 0.048 g SiO_2_ dosage has the highest Mr among all of sulfide-modified nZVI. According to the literature^49^, high Mr favors pollutants removal by ZVI through faster iron corrosion.

The hysteresis loops of particles with different nanoparticle seeding (Fig. 5B) also shows that magnetism of the synthesized materials can be affected by materials other than SiO_2_. The microstructure of materials, including crystalline state (crystal size, integrity, and homogeneity), conditions of grain boundary and stress, and bubble size and distribution can affect their magnetic properties^48^,^50^,^51^.

To verify our hypothesis that nanoparticle seeding can facilitate the formation of crystal Fe^0^ at high sulfidation (S/Fe molar ratio >0.28), we increased dithionite dosage from 0.6 g to 0.8 g, and repeated the syntheses without nanoparticle seeding and with 0.036 g nano-SiO_2_, TiO_2_ or Al_2_O_3_ seeding. As hypothesized, Fe^0^ formation and magnetization increased with nanoparticle seeding (Fig. S6).

### Mössbauer spectroscopy

**Figure 6:**
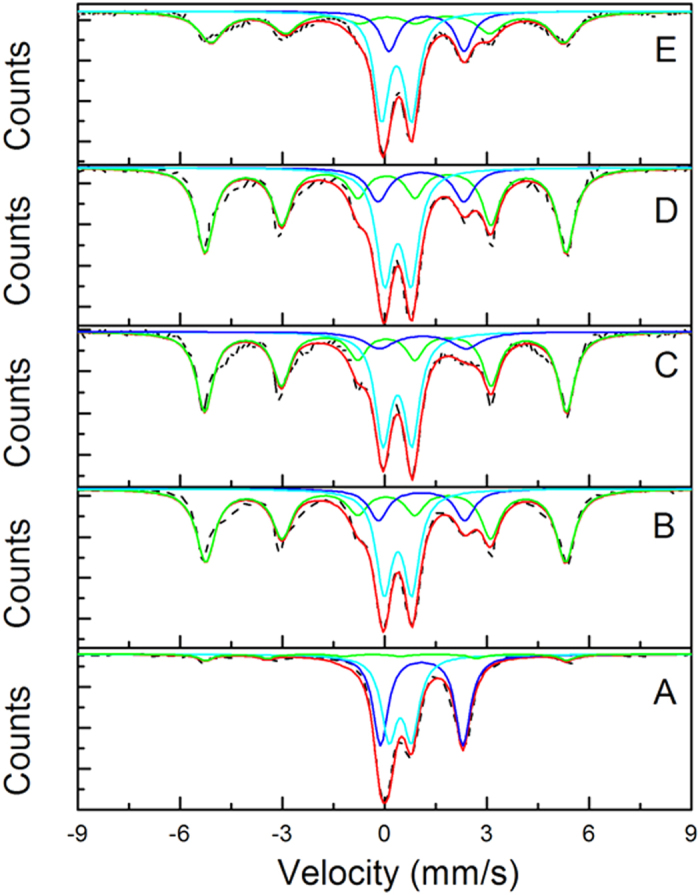
Room-temperature Mössbauer spectra of (**A**) S-nZVI (in glass), (**B**) FeSSi-0.012 g nano-SiO_2_, (**C**) FeSSi-0.048 g nano-SiO_2_, (**D**) S-nZVI-0.064 g nano-TiO_2_, and (**E**) S-nZVI-0.082 g nano-Al_2_O_3_. (- -, original line; 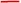, total fitting line; 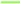, Fe^0^; 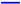, Fe^2+^; 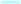, Fe^3+^;).

To further investigate the iron composition in the synthesized nanomaterials, Mössbauer spectroscopy was employed in this study (Fig. 6 and Table 4). A magnetic field or hyperfine field provides information on the electron spin density of a ^57^Fe nucleus in a magnetically ordered compound, and the isomer shift provides information on the oxidation state of Fe ions^52^. Every spectrum is composed of a sextet and two quadrupole doublets (Fig. 6). The sextet has a hyperfine field of about 33 T but no isomer shift, corresponding to zerovalent iron^53^. Among the two doublets, the one for Fe^2+^ is characterized by the large isomer shift, which is mainly due to the asymmetry of outer electrons; the other one is Fe^3+^, which has a smaller isomer shift due to the symmetric distribution of electrons on the *d* shell. As shown in Table 3, Fe^0^ accounts for 5.9% of total Fe in S-nZVI, 35.2% in FeSSi with 0.012 g nano-SiO_2_, 55.6% in FeSSi with 0.048 g nano-SiO_2_, 54.8% in S-nZVI with nano-TiO_2_, and 40.9% in S-nZVI with nano-Al_2_O_3_. Given the high Fe^0^ content in nZVI (~80%, shown by XANES), this result also corroborates our hypothesis that improved sulfidation suppresses Fe^0^ crystallization whereas nano-seeding can facilitate Fe^0^ formation in sulfidized systems. The accompanied change of ferrous and ferric ion content indicates the increased Fe^0^ content is due to the reduction of Fe^2+^ ions. Additionally, sulfidation can lead to the rise of isomer shift of Fe^2+^/Fe^3+^ ions, which may result from the decrease of shielding effect caused by *p, d*, and *f* shell on electrons in the *s* shell.

### Environmental applications

**Figure 7:**
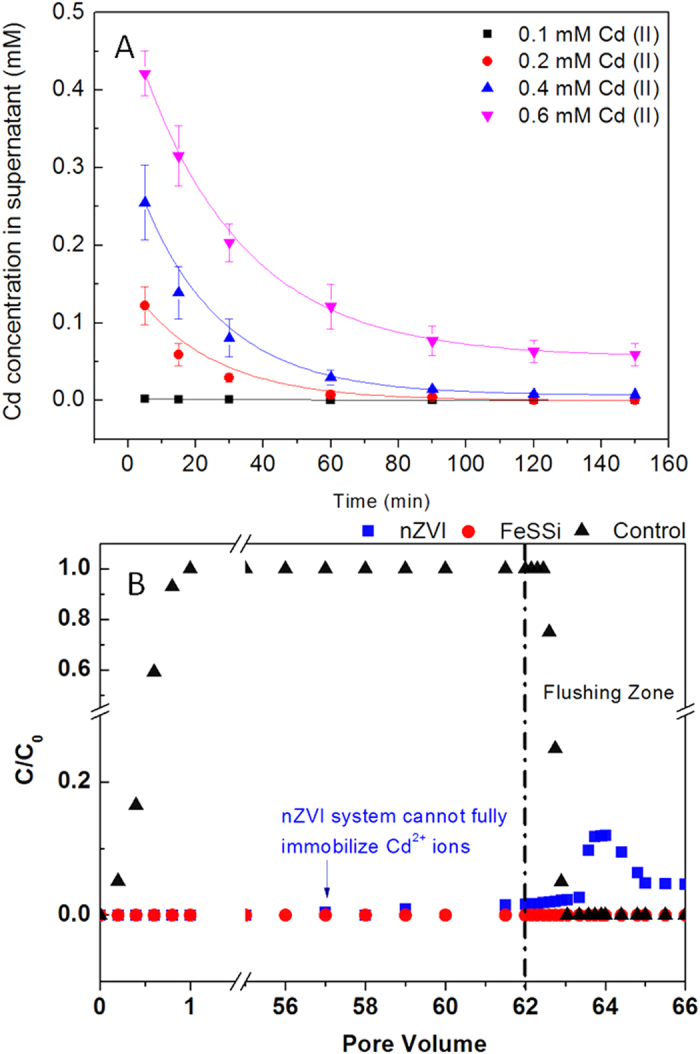
Cd^2+^ removal performance of FeSSi in batch experiments (**A**) and simulated permeable reactive barrier (PRB) (**B**) remediation experiment.

Our previous study showed that sulfidation of nZVI can improve its Cd^2+^ removal capacity^13^, but the magnetic properties of the synthesized particles decreases with increasing sulfidation. The loss of magnetism makes it difficult to perform magnetic solid-liquid separation, and thus makes it difficult to apply S-nZVI for water treatment. Through nanoparticles seeding, not only was sulfidation enhanced further, but magnetization was preserved. Cd^2+^ removal capacity of FeSSi was determined as 105 mg/g (Fig. 7A), which is much higher than that of nZVI (40 mg/g) and S-nZVI (80 mg/g)^13^. Chemical adsorption and precipitation are responsible for the Cd^2+^ immobilization. Additionally, FeSSi was also used to sequester Cu^2+^, Pb^2+^, Ni^2+^, Sb_2_O_7_^4−^ and Mo_2_O_7_^2−^ from artificial wastewater. After 2 hr reaction, in FeSSi system, the final concentration of metals were below the detection limit of ICP (Table S2), indicating that FeSSi is able to immobilize both metal cations and metal-oxo cluster anions.

To further study the chemical stability of Cd-FeSSi mixture, a long-term experiment was carried out in a 1-dimension sandbox to mimic the permeable reactive barrier system in groundwater remediation. As shown in the result presented as Fig. 7B, before flushing was carried out, Cd^2+^ ions was detected in the effluent from nZVI system from the 57th pore volume while it stayed undetectable in FeSSi system; Similarly, during flushing, a certain amount of Cd^2+^ ions became remobilized in nZVI system but not in FeSSi system. These results indicate that FeSSi is more applicable than nZVI for sequestering Cd^2+^ from groundwater due to its high removal capacity and the chemical stability of Cd-FeSSi mixture.

## Conclusions

In this study, we demonstrated a novel way to obtain a magnetic sulfide-modified nZVI, which is very effective for heavy metal removal from aqueous media. Sulfidation significantly improves the remediation capacity of nZVI for different classes of pollutants. However, it can also suppress Fe^0^ crystallization, leading to the decrease of Fe^0^ content (and thus, magnetic capacity) in the synthesized nanomaterial from 83.5% in nZVI to 5.9% in sulfide-modified nZVI. However, nano-seeding (to form FeSSi) can facilitate the formation of Fe^0^ crystals in sulfide-modified nZVI as confirmed by XRD and Mössbauer analyses. XANES analysis confirmed that nano-SiO_2_ seeding enhanced the final Fe^0^ content (to 55.6%) through increased reduction of both crystalline and amorphous iron oxide, an intermediate product during synthesis. While the synthesized magnetic FeSSi is covered by a significant amount of flake-like structures, spherical crystals are also observed. Thus, nanoparticle seeding can be used to enhance the magnetic properties of S-nZVI, which not only results in better magnetic solid-liquid separation, but also increases the formation of Fe^0^ crystals with high content of iron sulfide, resulting in a more effective nanomaterial for metal ion removal.

## Associated content

### Supporting information

Composition (Mass percentage) of nanoparticles collected from nZVI and FeSSi synthesis system as calculated by Linear Combination Fitting (Table S1); Removal percent of metals by S-nZVI and magnetic FeSSi nanoparticles (Table S2); Free energy of nucleation to explain the existence of 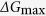 and 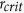
(Fig. S1); TEM image of the final material derived from system with 0.048 g nano-SiO2 dosage (Fig. S3); Energy-dispersive X-ray spectroscopy analysis for flake-like structure and particle area (Fig. 3B, stage III) (Fig. S4); Fourier transform magnitude of *K*^3^ weighted Fe *K*-edge EXAFS spectra of FeSSi collected at the middle (A) and last (B) stage of synthesis (Fig. S5). Hysteresis loop of different nanomaterials from systems with high dosage of dithionite (0.8 g) and different nanoparticle addition, namely nano-SiO_2_, nano-TiO_2_, nano-Al_2_O_3_ (Fig. S6).

## Experimental Section

### Reagent

Analytical grade sodium chloride (NaCl), sodium sulfate (Na_2_SO_4_) potassium sulfate (K_2_SO_4_), sodium dicarbonate (NaHCO_3_), calcium chloride (CaCl_2_), magnesium chloride (MgCl_2_), cadmium acetate (Cd(CH_3_COO)_2_), lead acetate (Pb(CH_3_COO)_2_), nickel chloride (NiCl_2_), copper chloride (CuCl_2_), potassium acid pyroantimonate (K_2_H_2_Sb_2_O_7_.4H_2_O), sodium selenate (Na_2_SeO_4_), ammonium dimolybdate ((NH_4_)_2_Mo_2_O_7_), sodium borohydride (NaBH_4_, 98%), dithionite (Na_2_S_2_O_4_), and ferric chloride hexahydrate (FeCl_3_.6H_2_O) were purchased from Sigma-Aldrich (Shanghai, China). 250 ml glass (GG-17) and plastic (poly(4-methyl-1-pentene), PMP) beaker were purchased from sinopharm. All chemicals were used without further purification. Deionized water was used for all reagent and particle suspension preparation.

### Influence of different amounts of nano-SiO_2_ (100 nm) on final nanomaterials

We prepared S-nZVI with and without nano-SiO_2_ seeding (100 nm, from Beijing DK nanotechnology Co. LTD). When seeded with nano-SiO_2_, the final particles were denominated FeSSi. Syntheses of nZVI and S-nZVI have been reported in our previous publications^13^. To prepare S-nZVI with nanoparticle seeding, 0.6 g dithionite was added into 100 ml of 3 g sodium borohydride solution (reductant). This solution was then seeded with different amounts of nano-SiO_2_ (0, 0.012, 0.024, 0.036, and 0.048 g), separately. After that, the mixture (continuously stirred by a magnetic stirrer) was titrated into 100 ml FeCl_3_.6H_2_O solution (3.84 g) at a titration rate of ~0.22 L/h, to obtain S-nZVI and different kinds of FeSSi. Aliquots were collected at time points during synthesis and analyzed for Fe^2+^ species via UV-Vis spectrometer (Biospec-1601, Shimadzu, Japan)^54^ after filtration (0.22 μm). Although sulfidation improves remediation capacity of nZVI, we showed in a previous study that S-nZVI particles were no longer magnetic above a S/Fe molar ratio of 0.28. This limited the extent of sulfidation that could be done without losing the magnetic behavior of the particles. We hypothesized that nanoparticle seeding would improve crystal formation of Fe^0^ so S/Fe molar ratios ≥ 0.28 were used in this study. To determine if reactor material played a role in nucleation/crystal formation, synthesis of S-nZVI was carried out as described in following section in either a plastic (PMP) or glass beaker.

### Influence of different nanoparticles on final nanomaterials

The effect of seeding with nano-TiO_2_ and nano-Al_2_O_3_ (both 100 nm, and from Beijing DK nanotechnology Co. LTD) instead of nano-SiO_2_ was also considered. To obtain equal molar concentrations as 0.048 g nano-SiO_2_, we used 0.064 g nano-TiO_2_ and 0.081 g nano-Al_2_O_3_. The synthesis procedure was the same as with nano-SiO_2_ seeding.

### Batch experiment for pollutants removal

The Cd removal capacity of the various materials synthesized (S-nZVI, FeSSi, nano-TiO_2_ or nano-Al_2_O_3_ seeded S-nZVI) particles was determined in a synthetic groundwater^55^. The synthetic groundwater was composed of 5 mM Cl^−^, 15 mM SO_4_^2−^, 3 mM HCO_3_^−^, 0.1 mM NO_3_^−^, 1 mM K^+^, 13.1 mM Na^+^, 10 mM Mg^2+^, and 2 mM Ca^2+^. 10 mM Cd^2+^ stock solution was used to prepare 50 ml 0.1, 0.2, 0.4, and 0.6 mM Cd^2+^ solution in polypropylene tubes. 0.5 ml stock mixtures of the various materials synthesized were added into separate solutions to achieve a concentration of 500 mg/L in each tube. All the tubes were placed on a shaker for 150 min. During the experiment, 1 ml aliquots were collected at 5, 15, 30, 60, 90, 120, and 150 min and separated into solid and liquid fractions using a magnet. Liquid fractions were diluted to 10 ml using 4% HNO_3_, and then analyzed via inductively coupled plasma (ICP, Agilent 720ES). Additional pollutant-removal studies were done to confirm the effectiveness of FeSSi for Cu^2+^, Pb^2+^, Ni^2+^, Sb_2_O_7_^4−^ and Mo_2_O_7_^2−^.

To evaluate the practicability of the nZVI/FeSSi in permeable reactive barrier (PRB) system for groundwater remediation, three one-dimension sandboxes (2 × 12 × 9 cm, with 2 × 2 × 9 cm PRB) were setup. Except the control treatment, inside PRB was the mixture of sands and nanomaterials (nZVI/S-nZVI, the wt% of nanomaterial is 10%); Out of PRB, the box was filled with pure sands. The influent is the synthetic groundwater with 3 mg/L Cd^2+^. We set the pore velocity at 0.5 cm/h and 1 pore volume equal to 24 hours. The effluent was collected and Cd^2+^ concentration was monitored continuously via ICP. After 62 pore volume, flushing was carried out to study the stability of Cd-nZVI or Cd-FeSSi mixture. To simulate the hostile grounwater condition, the influent was changed to an aerated groundwater with high concentration of Cl^−^ (50 mM) but without Cd^2+^ ions, and pH was adjusted to 6 by HCl (0.5 mM). The effluent was collected and Cd^2+^ concentration was measured by ICP.

All the tests were run in triplets, and the mean value was used in the figures.

### Instruments and analyses

Electron microscopy was performed using a JEOL JEM 2011 high-resolution TEM operated at 200 kV and equipped with a Hitachi S-3000N energy-dispersive X-ray spectrometer (EDS). Samples were prepared by depositing a drop of particles (suspended in 100% ethanol) onto a carbon-coated TEM grid in an anaerobic chamber. The samples were briefly exposed to air during transfer from the anaerobic chamber to the microscope. The water contact angle of the plastic and glass beaker was measured using a contact angle analysis instrument (OCA40, DataPhysics, Filderstadt, Germany). XRD was carried out using a Bruker D8 Advance X-ray diffraction instrument (Cu Kα). Diffraction angle (2*θ*) from 10° to 90° was scanned. VSM (Lake Shore 7410, USA) was used to study the magnetic properties of the derived final nanomaterials under room temperature. Coercivity, magnetization and retentivity were collected from the hysteresis loop. Mössbauer spectra were recorded at 298 K using a spectrometer with a triangular waveform and a source of ^57^Co (Lanzhou, China). The isomer shift; magnetic field; quadrupolar splitting and line width were refined using a least-squares fitting procedure in the Moss Winn program^56^. Fe K-edge XANES measurements were carried out on beamline BL 14W at Shanghai Synchrotron Radiation Facility (Shanghai, China). A Si(1 1 1) crystal monochromator was utilized to monochromatize the white beam. The storage ring energy was run at 3.5 GeV with injection currents of 200 mA. Before analyzing samples from experiments, the monochromator was calibrated through Fe foil measurement.

## Additional Information

**How to cite this article**: Su, Y. *et al*. Direct Synthesis of Novel and Reactive Sulfide-modified Nano Iron through Nanoparticle Seeding for Improved Cadmium-Contaminated Water Treatment. *Sci. Rep.* **6**, 24358; doi: 10.1038/srep24358 (2016).

## Supplementary Material

Supplementary Information

## Figures and Tables

**Table 1:** Fine structural parameters of FeSSi collected at different synthesizing stages analyzed by EXAFS.

**Sample**	**Atomic pairs**	**Bond length (**Å)	**Coordination number**	**R-factor**	**ΔE**_**0**_**(eV)**
FeSSi mid	Fe-O	1.99	6.6	0.0032	0.95
FeSSi final	Fe-Fe1	2.45	3.3	0.0013	−1.19
	Fe-Fe2	2.83	2.5	0.0013	−1.19
	Fe-O	1.89	1.2	0.0013	2.39
	Fe-S	2.40	0.8	0.0013	9

ΔE_0_ is the change in threshold energy.

**Table 2:** Crystal parameters from XRD analysis.

**Nanoparticles**	**Size of single Fe**^**0**^ **grain calculated by XRD (** 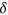 **, nm)**	**Crystallinity of Fe**^**0**^ ( 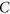 **, %)**
nZVI	96	73.7 (0)
S-nZVI in glass	324	11.2 (10.3)
S-nZVI in plastic	397	9.5 (22.2)
FeSSi-0.012g nSiO_2_	280	15.6 (9.0)
FeSSi-0.024g nSiO_2_	241	42.1 (6.0)
FeSSi-0.036g nSiO_2_	195	50.8 (5.2)
FeSSi-0.048g nSiO_2_	187	55.2 (5.0)
S-nZVI-0.064g nTiO_2_	195	35.4 (7.1)
S-nZVI-0.081g nAl_2_O_3_	191	33.8 (7.6)

Note: data in parentheses is crystallinity of NaCl in freeze-dried materials.

**Table 3:** Magnetism parameters from VSM tests.

**Synthesized materials**	**Coercivity Hc (Oe)**	**Magnetization M (emu/g)**	**Retentivity Mr (emu/g)**
nZVI	604.08	60.801	17.311
S-nZVI in glass	114.85	4.7179	0.11065
S-nZVI in plastic	100.94	2.2487	0.040582
FeSSi-0.012g SiO_2_	111.34	21.632	0.62875
FeSSi-0.024g SiO_2_	157.02	59.982	3.382
FeSSi-0.048g SiO_2_	180.77	65.459	4.2839
S-nZVI-0.064g TiO_2_	183.27	46.885	3.0347
S-nZVI-0.081g Al_2_O_3_	85.466	31.283	0.82309

**Table 4:** Mössbauer parameters of samples at room temperature.

**Parameters**	**S-nZVI (in glass)**	**FeSSi-SiO**_**2**_ **(0.012 g)**	**FeSSi-SiO**_**2**_ **(0.048 g)**	**S-nZVI -TiO**_**2**_	**S-nZVI -Al**_**2**_**O**_**3**_
Magnetic signal (Fe(0))					
C (%)	5.9	35.2	55.6	54.8	40.9
IS (mm/s)	−0.164	0.041	0.037	0.037	0.077
H (T)	32.940	32.898	32.985	32.916	32.122
Doublet signal (Fe(II))					
C (%)	49.8	16.5	9.9	12.7	18.0
IS(mm/s)	1.090	1.057	1.119	1.073	1.232
Doublet signal (Fe(III))					
C (%)	44.3	48.3	34.5	32.5	41.1
IS (mm/s)	0.458	0.397	0.377	0.387	0.350

Note: C, area percent; IS, isomer shift; H, magnetic field.
