# Corrigendum: Direct Synthesis of Novel and Reactive Sulfide-modified Nano Iron through Nanoparticle Seeding for Improved Cadmium-Contaminated Water Treatment

**DOI:** 10.1038/srep26918

**Published:** 2016-06-01

**Authors:** Yiming Su, Adeyemi S. Adeleye, Yuxiong Huang, Xuefei Zhou, Arturo A. Keller, Yalei Zhang

Scientific Reports 6: Article number: 24358; 10.1038/srep24358 published online: 04202016; updated: 06012016.

There is a typographical error in Table 2:

“Size of single Fe^0^ grain calculated by XRD (δ, nm)”.

should read:

“Size of single Fe^0^ grain calculated by XRD (δ, Å)”.

