# Supplementary material for: Direct Synthesis of Novel and Reactive Sulfide-modified Nano Iron through Nanoparticle Seeding for Improved Cadmium-Contaminated Water Treatment
Source: Sci Rep. 2016 Apr 20;6:24358. doi: 10.1038/srep24358 (PMC4837343; doi:10.1038/srep24358)
Supplement: Supplementary Information [file srep24358-s1.docx]

Supplementary Information

**Direct Synthesis of Novel and Reactive Sulfide-modified Nano Iron through Nanoparticle Seeding for Improved Cadmium-Contaminated Water Treatment**

Yiming Su^a^, Adeyemi S. Adeleye^b,c^, Yuxiong Huang^b^, Xuefei Zhou^a^, Arturo A. Keller^b,c＊^, Yalei Zhang^a,d,＊＊^

^a^*State Key Laboratory of Pollution Control and Resources Reuse, Tongji University, Shanghai 200092, China*.

^b^*Bren School of Environmental Science & Management, University of California, Santa Barbara, 3420 Bren Hall, CA 93106, USA.*

^c^*University of California Center for Environmental Implications of Nanotechnology, Santa Barbara, California, USA.*

^d^Key Laboratory of Yangtze Water Environment for Ministry of Education, Tongji University, Shanghai 200092, China.

**Table caption:**

Table S1. Composition (Mass percentage) of nanoparticles collected from nZVI and FeSSi synthesis system as calculated by Linear Combination Fitting.

Table S2 Removal percent of metals by S-nZVI and magnetic FeSSi nanoparticles

**Figure captions:**

Figure S1. Fe^2+^ trend in FeCl_3_•6H_2_O solution during titration with different nano materials addition (all the collected samples were passed through a 0.22 um filter);

Figure S2: TEM image of the final material derived from system with 0.048 g nano-SiO_2_ dosage;

Figure S3: Energy-dispersive X-ray spectroscopy analysis for flake-like structure and particle area (Fig. 3B, stage III);

Figure S4: Free energy of nucleation to explain the existence of $\Delta G_{\max}$ and $r_{crit}$;

Figure S5: Fourier transform magnitude of *K*^3^ weighted Fe *K*-edge EXAFS spectra of FeSSi collected at the middle (A) and last (B) stage of synthesis.

Figure S6: Hysteresis loop of different nanomaterials from systems with high dosage of dithionite (0.8 g) and different nanoparticle addition, namely nano-SiO_2_, nano-TiO_2_, nano-Al_2_O_3_ .

Table S1. Composition (Mass percentage) of nanoparticles collected from nZVI and FeSSi synthesis system as calculated by Linear Combination Fitting

| Sample ID | Fe(0) | FeO | gamma Fe_2_O_3_ | Fe_3_(PO_4_)_2_ | FeS | FeSO_4_ | R-factor |
| --- | --- | --- | --- | --- | --- | --- | --- |
| FeSSi mid | - | - | 11.7 | 63.7 | 5.0 | 18.7 | 0.000083 |
| FeSSi Final | 55.6 |  | - | 21.0 | 24.2 | - | 0.000179 |
| Fe mid | 75.6 | 16.2 | 8.8 | - | - | - | 9.75E-05 |
| Fe final | 83.5 | 9.3 | 7.8 | - | - | - | 0.000284 |

Table S2 Removal percent of metals by S-nZVI and magnetic FeSSi nanoparticles

| Metal | Percent removed by S-nZVI (%) | Percent removed by FeSSi (%) |
| --- | --- | --- |
| Cu^2+^ | 98.4 | - |
| Pb^2+^ | 96.9 | - |
| Ni^2+^ | 90.6 | - |
| Sb_2_O_7_^4-^ | 89.8 | - |
| Mo_2_O_7_^2-^ | 83.2 | - |

Note: “-” means below the detection limit of ICP.

To see how effective the particles are for removing other heavy metals in artificial industrial wastewater (30 mM Cl^-^, 15 mM SO_4_^2-^, 10 mM K^+^, 30 mM Na^+^ and 10 mM Mg^2+^), a certain volume of S-nZVI (with 0.4 g dithionite addition during synthesizing) and FeSSi stock suspensions was added into six separate heavy metal solutions (Ni^2+^, Cu^2+^, Pb^2+^, Sb_2_O_7_^4-^ and Mo_2_O_7_^2-^, 50 mg/L), to achieve a concentration of 500 mg/L. The total volume of the mixture was 50 ml, leaving minimal headspace in the 50 mL polypropylene tubes. After 2 hr shaking, magnet-facilitated solid-liquid separation was performed, and 1 ml of supernatant was collected and analyzed via ICP for heavy metals.


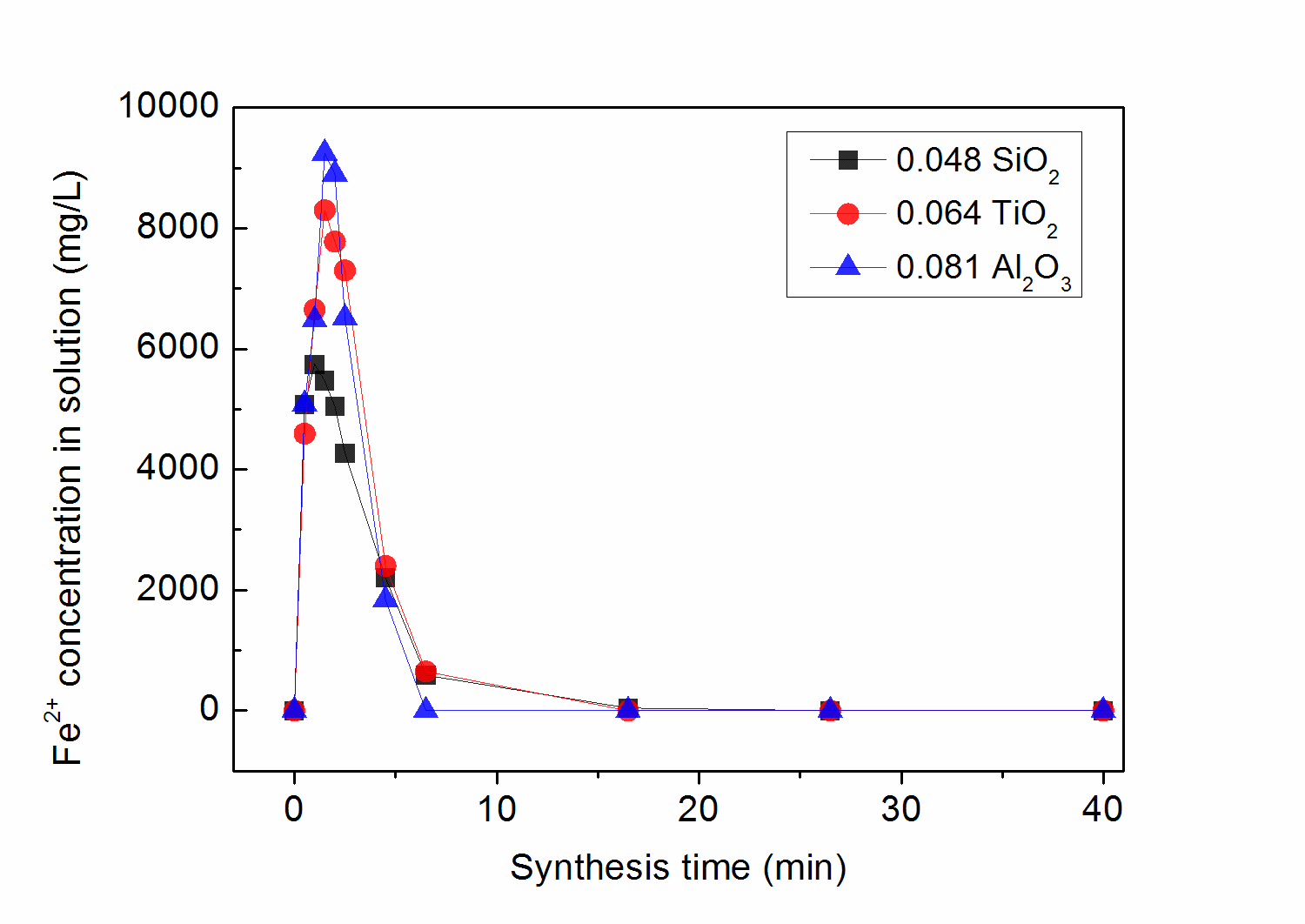


Figure S1. Fe^2+^ trend in FeCl_3_•6H_2_O solution during titration with different nano materials addition (all the collected samples were passed through a 0.22 um filter.


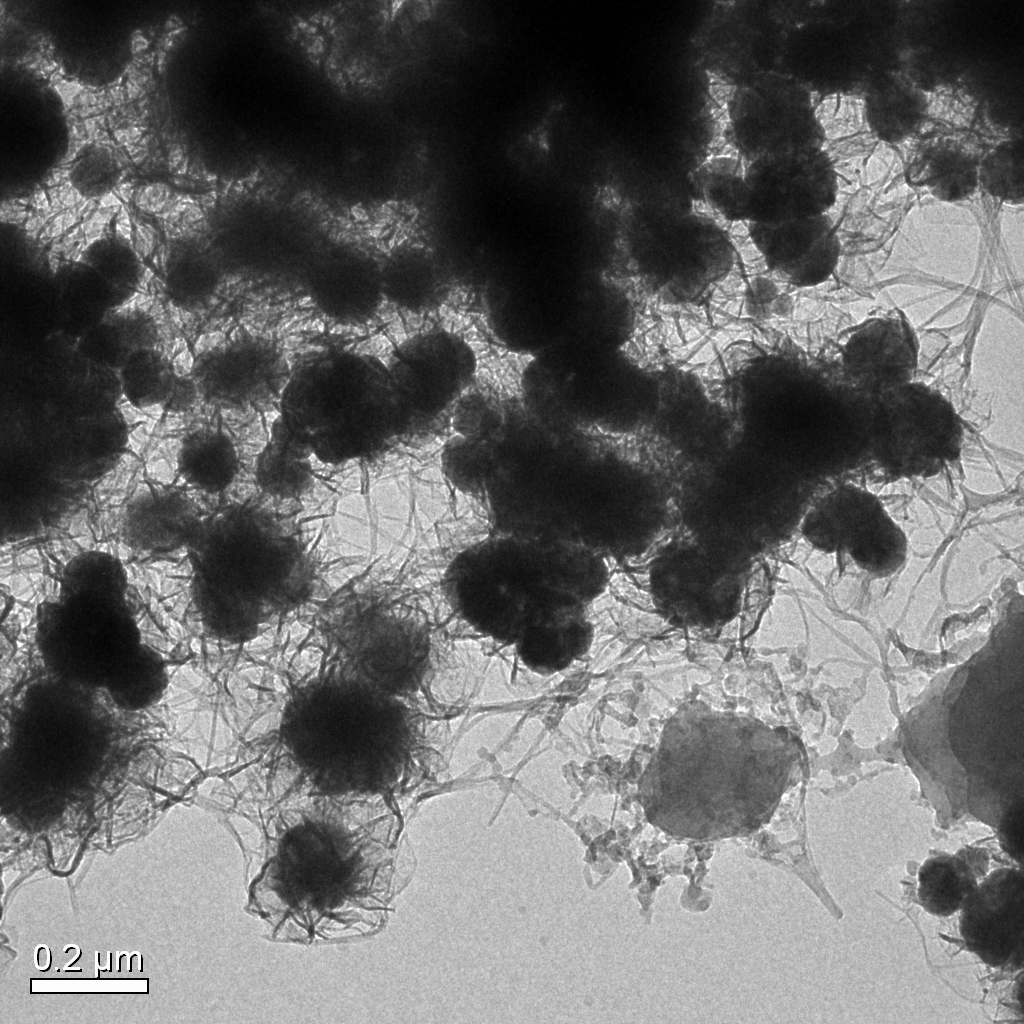


Figure S2. TEM image of the final material derived from system with 0.048 g SiO_2_ addition.

flake

Figure S3. Energy-dispersive X-ray spectroscopy analysis for flake-like structure and particle area (Fig. 3b, stage III).


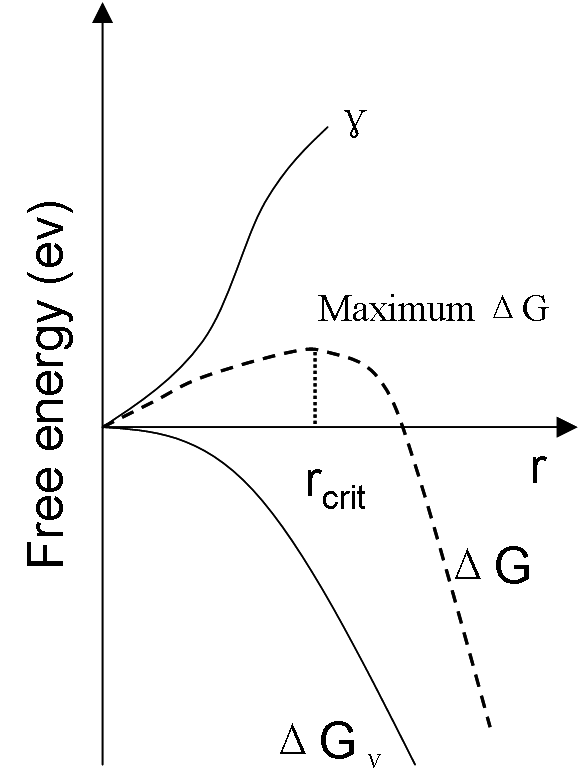


Figure S4: Free energy of nucleation to explain the existence of $\Delta G_{\max}$ and $r_{crit}$;


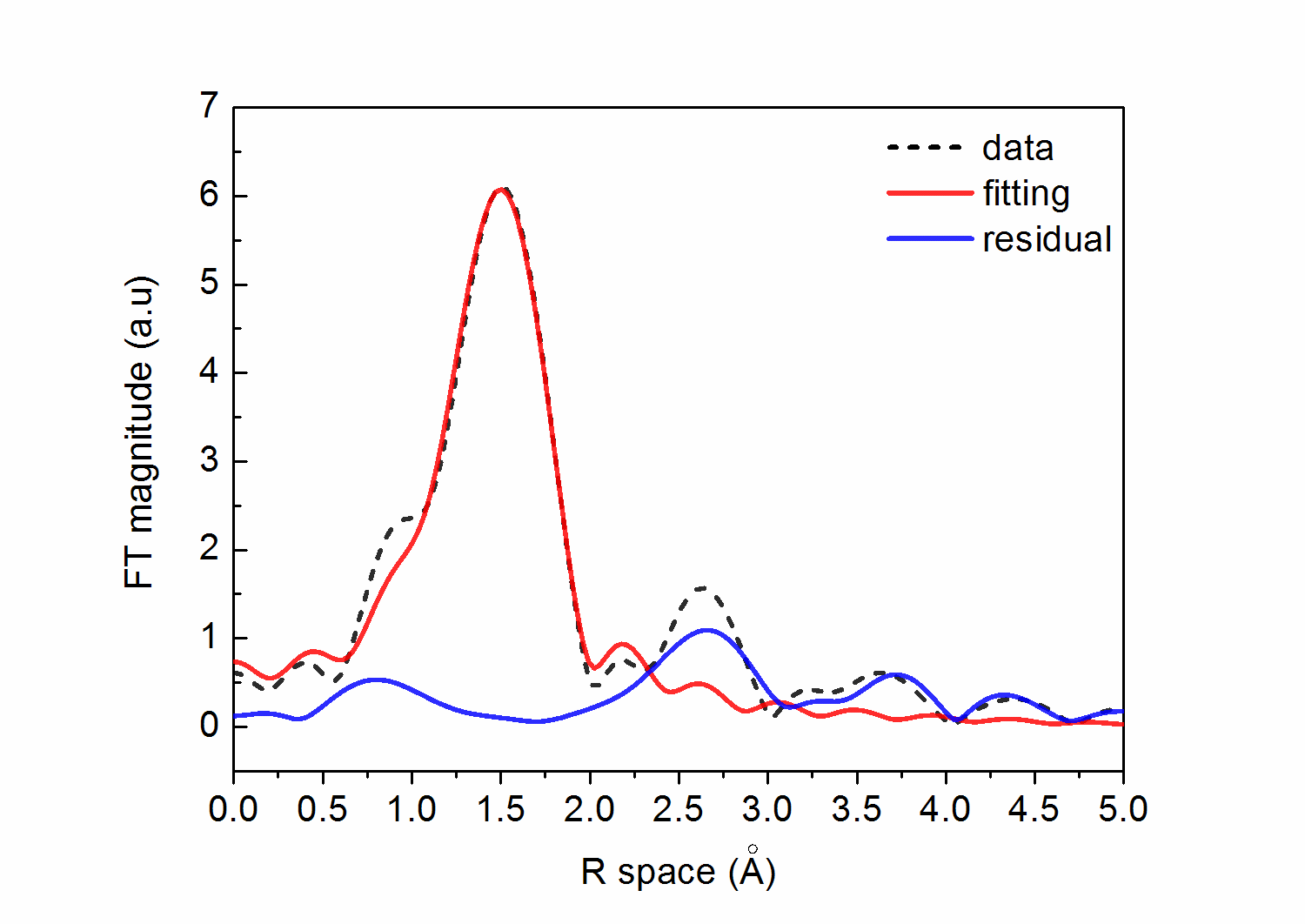


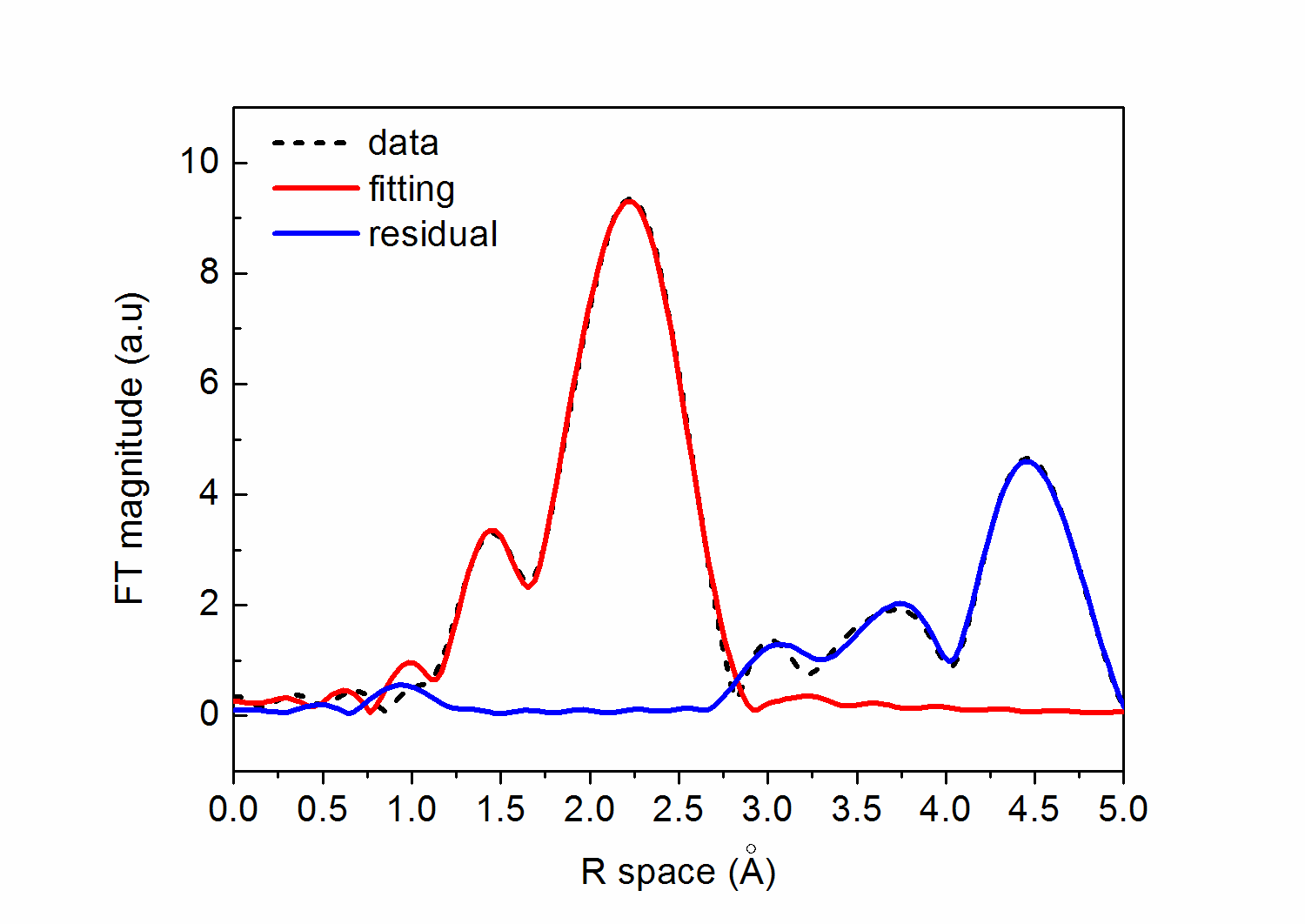


(B)

(A)

Figure S5. Fourier transform magnitude of *K*^3^ weighted Fe *K*-edge EXAFS spectra of FeSSi collected at the middle (A) and last (B) stage of synthesis.


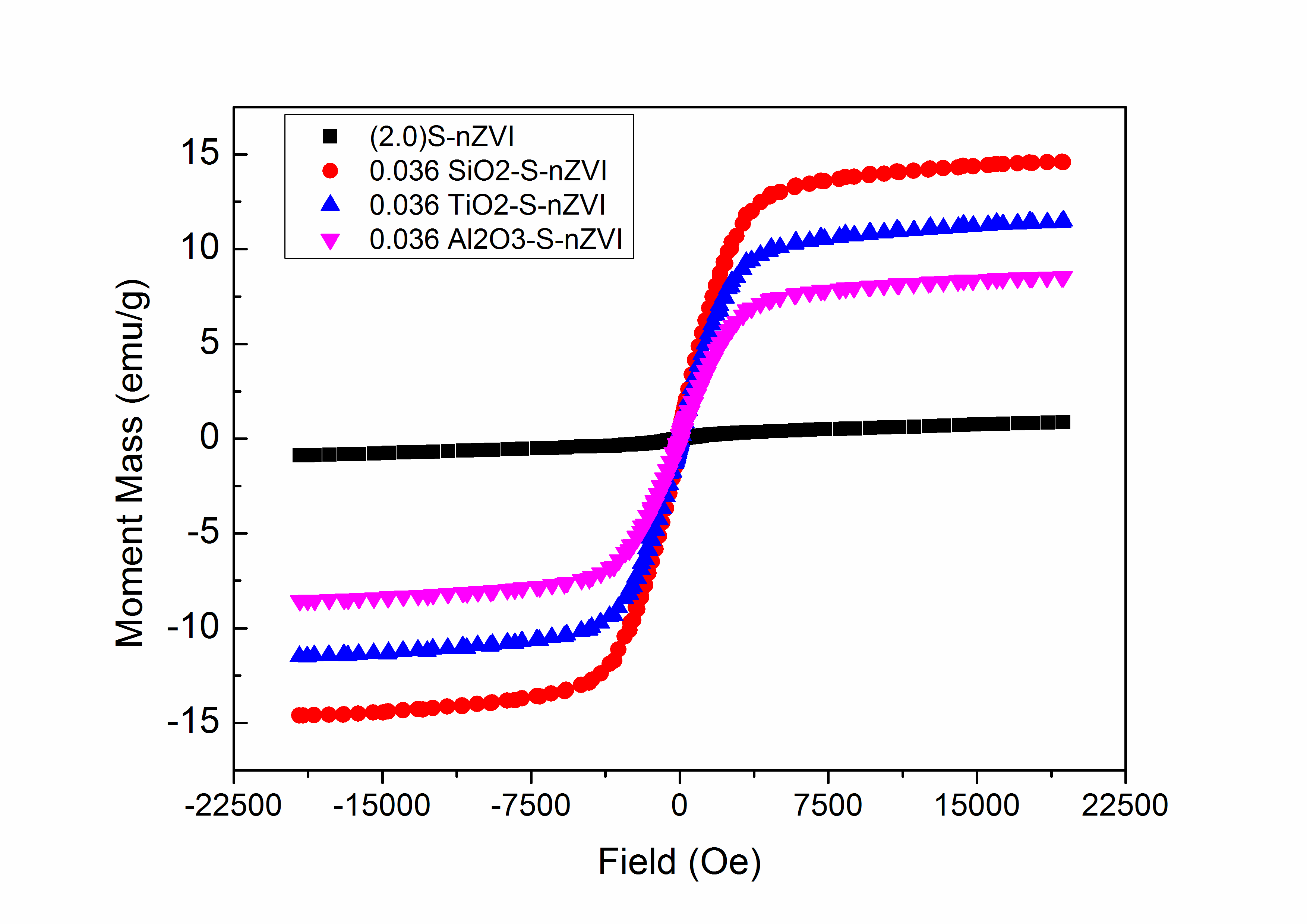


Figure S6. Hysteresis loop of different nanomaterials from systems with high dosage of dithionite (0.8 g) and different nanoparticle addition, namely nano-SiO_2_, nano-TiO_2_, nano-Al_2_O_3_
